# Macrophage recruitment and epithelial repair following hair cell injury in the mouse utricle

**DOI:** 10.3389/fncel.2015.00150

**Published:** 2015-04-22

**Authors:** Tejbeer Kaur, Keiko Hirose, Edwin W. Rubel, Mark E. Warchol

**Affiliations:** ^1^Department of Otolaryngology, Washington University School of Medicine, St. Louis, MOUSA; ^2^The Virginia Merrill Bloedel Hearing Research Center and Department of Otolaryngology – Head and Neck Surgery, University of Washington School of MedicineSeattle, WA, USA

**Keywords:** vestbular, auditory, hair cell, neuroimmunology, ototoxicity

## Abstract

The sensory organs of the inner ear possess resident populations of macrophages, but the function of those cells is poorly understood. In many tissues, macrophages participate in the removal of cellular debris after injury and can also promote tissue repair. The present study examined injury-evoked macrophage activity in the mouse utricle. Experiments used transgenic mice in which the gene for the human diphtheria toxin receptor (huDTR) was inserted under regulation of the Pou4f3 promoter. Hair cells in such mice can be selectively lesioned by systemic treatment with diphtheria toxin (DT). In order to visualize macrophages, Pou4f3–huDTR mice were crossed with a second transgenic line, in which one or both copies of the gene for the fractalkine receptor CX3CR1 were replaced with a gene for GFP. Such mice expressed GFP in all macrophages, and mice that were CX3CR1^GFP/GFP^ lacked the necessary receptor for fractalkine signaling. Treatment with DT resulted in the death of ∼70% of utricular hair cells within 7 days, which was accompanied by increased numbers of macrophages within the utricular sensory epithelium. Many of these macrophages appeared to be actively engulfing hair cell debris, indicating that macrophages participate in the process of ‘corpse removal’ in the mammalian vestibular organs. However, we observed no apparent differences in injury-evoked macrophage numbers in the utricles of CX3CR1^+/GFP^ mice vs. CX3CR1^GFP/GFP^ mice, suggesting that fractalkine signaling is not necessary for macrophage recruitment in these sensory organs. Finally, we found that repair of sensory epithelia at short times after DT-induced hair cell lesions was mediated by relatively thin cables of F-actin. After 56 days recovery, however, all cell-cell junctions were characterized by very thick actin cables.

## Introduction

Hair cells are the sensory receptors of the inner ear. They detect sound vibrations and head movements, yielding information that is transmitted to the brain by auditory and vestibular afferent neurons. Within the various sensory epithelia of the ear, hair cells are completely surrounded by so-called supporting cells, which provide structural and trophic support to both hair cells and afferent neurons. Hair cells can be injured or lost as a result of noise exposure, ototoxicity, or as consequence of normal aging. The loss of hair cells can create cellular breaches within the sensory epithelia of the ear, leading to mixing of the inner ear fluids, and disruption of ionic homeostasis. As such, it is critical that such lesions are quickly repaired, and prior studies have shown that the loss of hair cells triggers adjoining supporting cells to undergo a coordinated and active cellular program, leading to removal of hair cell debris, and reformation of cell-cell junctions at the lumenal surface (e.g., Raphael and Altschuler, 1991; Meiteles and Raphael, 1994; Hordichok and Steyger, 2007; Bird et al., 2010; Anttonen et al., 2014).

Although most tissues and organ systems possess intrinsic cellular repair mechanisms, response to injury can also involve resident and recruited macrophages. These cells respond to injury by removing the debris of dead cells and can also secrete growth factors that evoke cell division and/or migration (Stefater et al., 2011). Prior studies have shown that the inner ear contains resident populations of macrophages and that hair cell injury leads to macrophage activation and recruitment (Warchol, 1997; Hirose et al., 2005; Warchol et al., 2012). However, the precise role of macrophages in the injured ear remains unknown. In the present study, we used novel transgenic mouse models to study the behavior of inner ear macrophages after selective ablation of vestibular hair cells. Our data indicate that the mouse utricle contains a resident population of macrophages, which are mainly confined to the stromal tissue below the sensory epithelium. Hair cell injury causes macrophages to enter the sensory epithelium, and some of those recruited cells appear to phagocytose hair cell debris. The signal(s) that promote macrophage recruitment are not known, but our data further suggest that signaling via fractalkine (CX3CL1/CX3CR1) is not required for macrophage entry into the injured vestibular epithelium. Notably, we also observed filamentous actin ‘basket’ structures within the injured epithelia, similar to those that have been shown to engulf hair cell debris in the avian utricle (Bird et al., 2010). Together, these results suggest that macrophages play an active role in the removal of dying hair cells, but that supporting cells may also contribute to this process.

## Materials and Methods

### Animals

All experimental protocols involving animals were approved by the Animal Studies Committee of the Washington University School of Medicine. Both of the transgenic mouse lines used in these studies have been described in previous publications. The Pou4f3–huDTR mouse line was generated by insertion of the gene for the human form of the diphtheria toxin receptor (huDTR, also known as HB-EGF) under regulation of the promoter for the Pou4f3 transcription factor. This factor, which is also known as Brn3.1 and Brn3c, is expressed only in developing and mature hair cells (Erkman et al., 1996; Xiang et al., 1997). As such, these mice express huDTR in hair cells and systemic administration of diphtheria toxin (DT) results in the selective death of hair cells in both the cochlea and vestibular organs (Tong et al., 2011, 2015; Golub et al., 2012; Mahrt et al., 2013; Cox et al., 2014).

C57BL6/J mice that were heterozygous for the Pou4f3–huDTR transgene were crossed with a second transgenic line in which one or both alleles of the gene for the fractalkine receptor (CX3CR1) was replaced with cDNA for GFP (provided by Dan Littmann, New York University, NY, USA). These mice express GFP in macrophages, monocytes, microglia, NK cells, and some T cells (Jung et al., 2000). In addition, CX3CR1^GFP/+^ mice retain one copy of the gene for the fractalkine receptor and respond normally to the fractalkine ligand, while mice that are CX3CR1^GFP/GFP^ lack this receptor and are not responsive to fractalkine. Experimental mice were either *CX3CR1*^+/GFP^*:Pou4f3*^DTR/+^ or *CX3CR1*^GFP/GFP^*:Pou4f3*^DTR/+^, while control mice lacked the Pou4f3–huDTR transgene but were heterozygous or homozygous for CX3CR1-GFP (*CX3CR1*^+/GFP^*:Pou4f3*^+/+^ or *CX_3_CR1*^GFP^*:Pou4f3*^+/+^). Mice were housed in the animal facilities of the Division for Comparative Medicine at Washington University. They were kept on a 12 hr/day night light cycle and had free access to food and water.

### Creation of Hair Cell Lesions

Adult mice (6–8 weeks) of either sex received a single intramuscular (i.m.) injection of (DT, 25ng/gm, Sigma-Aldrich). Mice then received daily intraperitoneal injections of lactated Ringer’s solution (0.5 ml, for 3–6 days), and mouse food was supplemented with high-calorie gel (Tomlyn from Nutri-Cal). Mice were allowed to recover for 7, 14, or 56 days, at which time they were deeply anesthetized with Somnasol (0.1 ml/20 gm), and perfused transcardially with 4% paraformaldehyde in 0.1 M phosphate buffer (Electron Microscopy Sciences). Temporal bones were isolated and decalcified by treatment for 48–72 h in 0.1 M EDTA (at 4∘C).

### Histological Methods

Utricles were dissected from the decalcified temporal bones and rinsed in PBS. Non-specific antibody binding was reduced by treatment for 2 hr in PBS with 5% normal horse serum (NHS) and 0.2% Triton X-100. Specimens were incubated overnight at room temperature in primary antibodies. Hair cells were labeled with antibody against Myosin VI (goat polyclonal, Santa Cruz Biotechnology, 1:100) or otoferlin (mouse monoclonal, Abcam, 1:200). Intensity of macrophage-expressed GFP was amplified via labeling with an antibody against GFP (rabbit, Invitrogen, 1:500). After labeling with primary antibodies, specimens were thoroughly rinsed in PBS and incubated for 2 hr in secondaries (Alexa 546-anti-mouse or Alexa 546 anti-goat, and Alexa 488 anti-rabbit – Invitrogen). All specimens were also stained with Alexa-647 conjugated phalloidin (Invitrogen) and cell nuclei were labeled with DAPI. After labeling, specimens were mounted on microscope slides in glycerol/PBS (9:1), and coverslipped.

### Cellular Imaging and Analysis

All specimens were imaged using a Zeiss LSM 700 confocal microscope. Volocity 3D image analysis software (Version 6.1.1, PerkinElmer) was used to reconstruct and process the images. Quantitative data were collected directly from these images and are presented as mean ± SD. Statistical tests were carried-out using Microsoft Excel software.

## Results

### Hair Cell Loss Following Diphtheria Toxin Treatment

**FIGURE 1:**
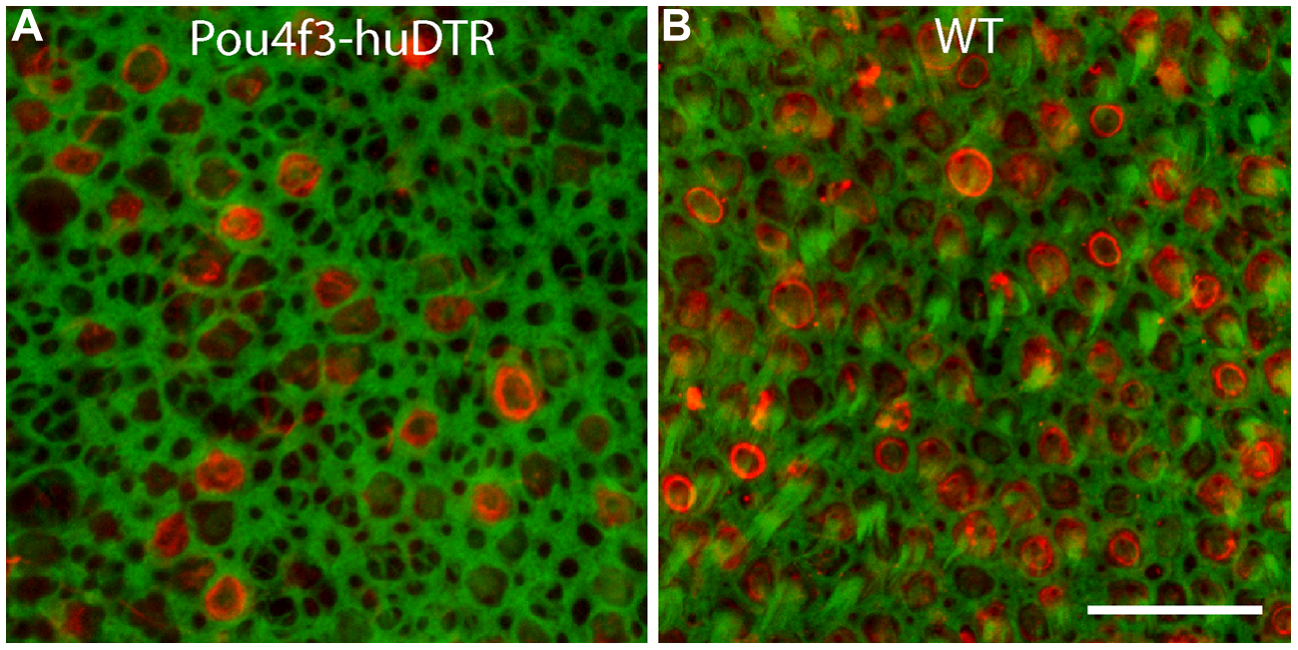
**Systemic treatment with diphtheria toxin (DT) lesions hair cells in the utricles of Pou4f3–huDTR (human form of the diphtheria toxin receptor) transgenic mice.** Knock-in mice in which one copy of the gene for Pou4f3 was replaced with the gene for huDTR received a single 25 ng/gm injection of DT. After 7 days, mice were perfused and utricles were immunolabeled for myosin VI (red, hair cells) and also stained with phalloidin (green). A partial loss of hair cells was observed in the utricles of Pou4f3–huDTR mice **(A)**, when compared with littermates that did not possess the Pou4f3–huDTR transgene, but were also treated with DT **(B)**. Scale bar = 20 μm.

All mice received a single 25 ng/gm injection of DT and were allowed to survive for 7, 14, or 56 days. Utricles of mice that possessed the Pou4f3–huDTR transgene displayed a significant hair cell lesion at 7 days after the DT injection (**Figure 1**). Hair cell loss was assessed by quantification of cells that displayed myosin VI or otoferlin immunoreactivity and also possessed phalloidin-labeled stereocilia bundles (**Figure 1**). Such cells were counted from six 50 μm × 50 μm regions, located in the central portion of the sensory epithelium, and the resulting values were normalized to hair cells/10,000 μm^2^. Utricles from Pou4f3–huDTR mice contained 46.0 ±_12.8 HC/10,000 μm^2^ (*n* = 7 utricles). In contrast, utricles from non-transgenic control mice (C57/Bl6 strain) appeared unaffected by DT treatment and contained 224.8 ± 16.0 hair cells/10,000 μm^2^ (*n* = 6 utricles). Finally, utricles from Pou4f3–huDTR mice fixed at 14 days after DT injection contained 29.6 ± 10.4 hair cells/10,000 μm^2^ (*n* = 7 utricles). These data indicate that a single injection of DT causes the death of ∼85% of the hair cell population of the utricle within 14 days.

### Remodeling of Epithelial Junctions Following Hair Cell Injury

**FIGURE 2:**
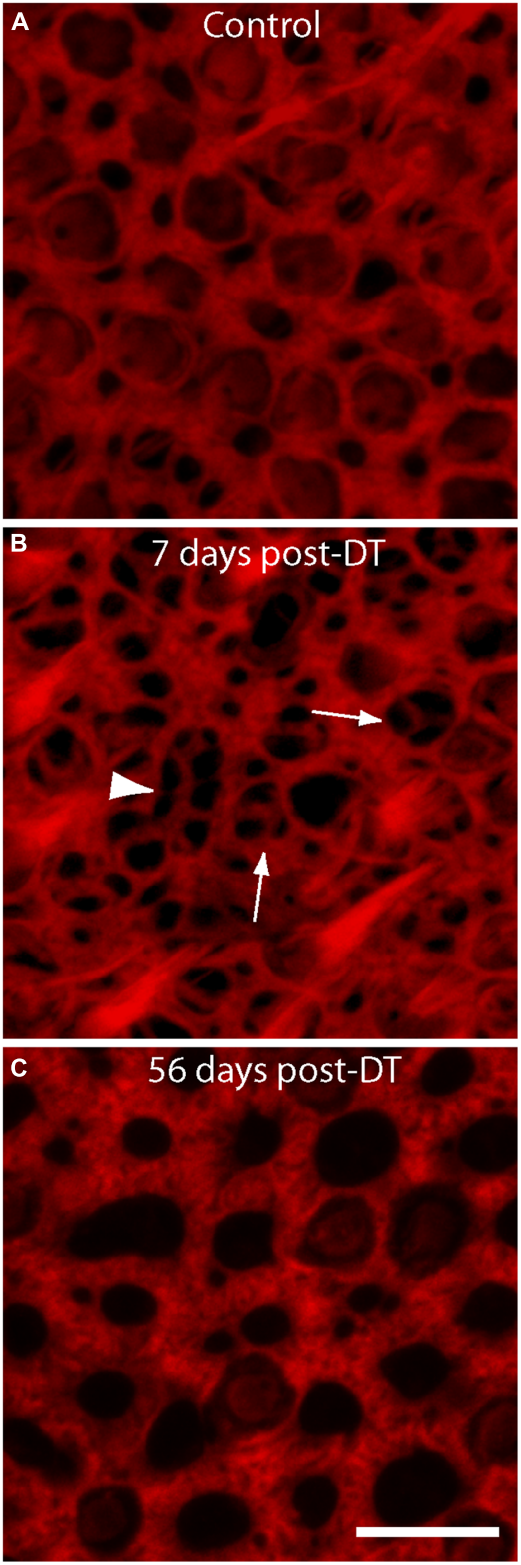
**Changes in the structure of actin bundles at cell–cell junctions following DT-mediated hair cell loss.** All specimens were stained with phalloidin (red), which labels filamentous actin. **(A)** Junctions in undamaged utricles of control mice were comprised of thick actin bundles. Numerous stereocilia bundles were also apparent. **(B)** At 7 days after DT-induced hair cell injury, the epithelia contained many thin actin fibers, which are likely to be loci of epithelial repair after hair cell loss. Structures of thinner actin usually occupied a single cell-width (arrows), but some structures appeared to extend for several cell widths (arrow head). **(C)** Sensory epithelia of utricles examined at 56 days recovery from hair cell lesions contained very thick actin cables, suggesting nearly complete repair of the lumen. Scale bar = 10 μm.

The sensory epithelia of the inner ear form barriers between two fluid spaces of highly differing ionic composition (endolymph and perilymph). Repair of this epithelial barrier after injury or hair cell loss is critical to the maintenance of these fluids. Injury to the vestibular epithelia of non-mammals leads to rapid reformation of cell-cell junctions by surviving supporting cells (e.g., Hordichok and Steyger, 2007; Bird et al., 2010). However, cellular junctions in the vestibular maculae of mammals are supported by thick bundles of filamentous actin (e.g., Burns et al., 2008), which may limit their ability to quickly reseal cellular lesions. In order to explore this issue, we examined the structure of actin cables at the lumenal surfaces of utricles after DT-induced hair cell loss. Specimens that were fixed at 7 days post-DT contained numerous thin actin bundles (**Figure 2**, arrows). Many of these were in the form of three radially directed thin actin bands (**Figure 2**, arrowhead). Such formations were previously described by Burns and Corwin (2014) and are likely to reflect epithelial repair after the loss of a single hair cell. However, we also noted more complex structures, which were comprised of thin actin bands that extended for several cell widths (arrow head, **Figure 2B**). These structures may have been created after the loss of 2–3 adjoining hair cells. Notably, at 56 days post-DT, the sensory epithelia appeared uniform and cell–cell junctions were characterized by very thick actin bundles (**Figure 2C**).

### Hair Cell Loss Attracts Macrophages into the Sensory Epithelium

**FIGURE 3:**
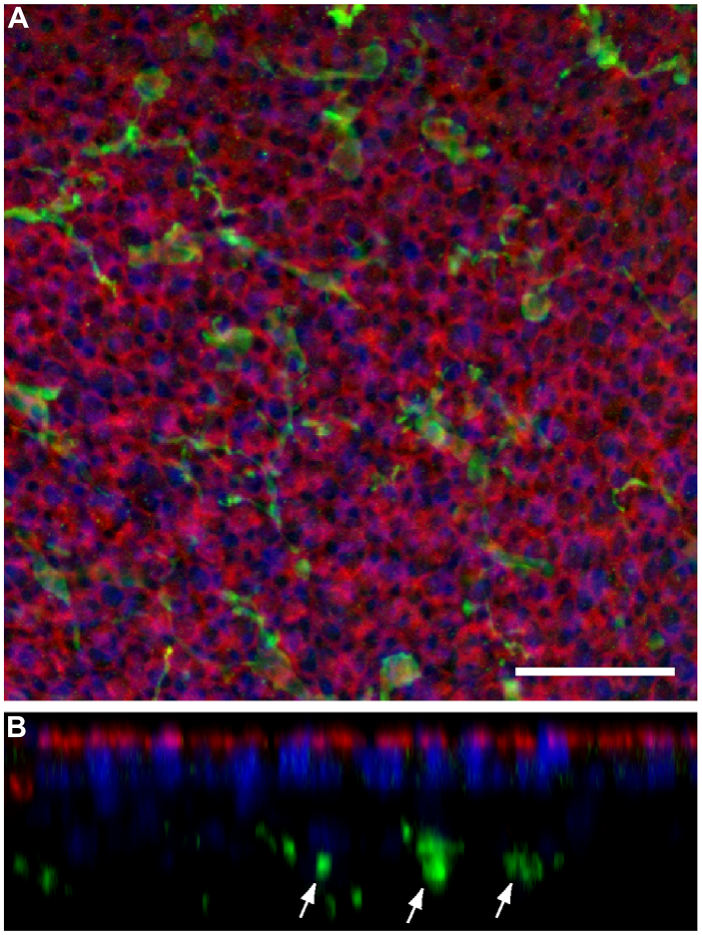
**Normal utricles contain resident populations of macrophages, which are primarily confined to the stromal tissue below the sensory epithelium. (A)** Maximum intensity projection of a confocal stack collected from the central region of a histologically processed mouse utricle, to a depth of 20 μm. **(B)** Computationally reconstructed ‘z-slice,’ showing a slide view of the same image. Cell-cell junctions are labeled with phalloidin (red) and cell nuclei are stained with DAPI (blue). In addition, GFP-expressing macrophages (green) appear distributed throughout the specimen. However, examination of z-slice images reveals that nearly all macrophages reside in the stromal tissue, below the sensory epithelium (arrows). Scale bar = 30 μm.

**FIGURE 4:**
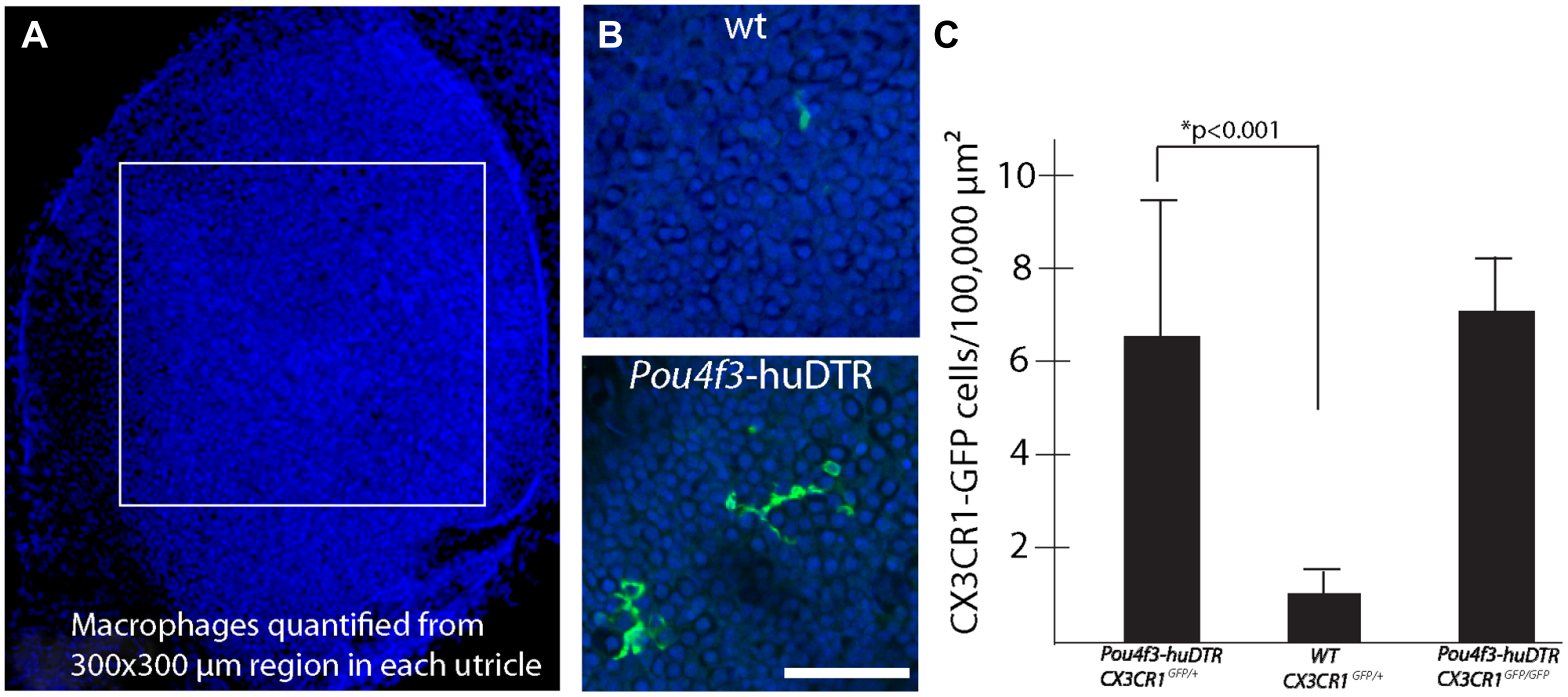
**Hair cell lesion increases the numbers of macrophages in the sensory epithelia of mouse utricles. (A)** Confocal image stacks were obtained from 300 μm × 300 μm regions in the central portion of mouse utricles. **(B)** Examination of the DAPI-labeled nuclei of hair cells and supporting cells (blue) was used to determine the vertical extent of the sensory epithelium and the total number of GFP-labeled macrophages (green) within the sensory region was quantified. **(C)** Quantitative data revealed that injection of DT into Pou4f3–huDTR mice resulted in increased numbers of macrophages within the sensory epithelium, compared to normal C57/Bl6 mice that also received DT (*p < 0.001). However, the numbers of macrophages in the sensory epithelia of DT-treated Pou4f3–huDTR–CX3CR1^GFP/+^ mice was nearly identical to that observed in the sensory epithelia of Pou4f3–huDTR–CX3CR1^GFP/GFP^ mice.

Prior studies have shown that the auditory organs of both birds and mammals contain resident populations of macrophages (e.g., Warchol, 1997; Hirose et al., 2005; Warchol et al., 2012), and macrophages are also present in the vestibular organs of mice (Zhang et al., 2013). Examination of undamaged utricles from CX3CR1^GFP/+^ or CX3CR1^GFP/GFP^ mice revealed numerous macrophages, which were mainly confined to the stromal tissue that underlies the sensory epithelium (**Figure 3**). In order to determine whether macrophages were recruited toward sites of hair cell injury, we quantified GFP-positive macrophages in the sensory epithelium of utricles from both normal and DT-lesioned animals. Confocal image stacks were obtained from whole-mount utricle specimens, and the numbers of macrophages within the sensory epithelium (i.e., within the same image planes as the DAPI-stained nuclei of hair cells and supporting cells) was quantified from a single field of 300 μm × 300 μm, located in the center of the utricle (**Figure 4A**). Resulting density data were then normalized to macrophages/100,000 μm^2^. At 7 days after DT treatment, utricles from Pou4f3–huDTR–CX3CR1^GFP/+^ mice contained 6.5 ± 3.2 GFP-labeled macrophages/100,000 μm^2^ (*n* = 8), while utricles from DT-treated CX3CR1^GFP/+^ mice that lacked the Pou4f3–huDTR transgene contained 1.0 ± 0.7 macrophages/100,000 μm^2^ (*n* = 8, *p* < 0.001; **Figures 4B,C**). These findings indicate that the loss of hair cells leads to the recruitment of macrophages into the sensory epithelium. However, further quantification of epithelial macrophage numbers suggests that this increase is transitory. Specifically, at 14 days post-DT, utricles from Pou4f3–huDTR mice contained 1.9 ± 1.1 macrophages/100,000 μm^2^ (*n* = 7 total: 2 CX3CR1^GFP/+^ and 5 CX3CR1^GFP/GFP^), while utricles from littermate mice that lacked the Pou4f3–huDTR transgene contained 1.4 ± 0.6 macrophages/100,000 μm^2^ (*n* = 4 total: 1 CX3CR1^GFP/+^ and 3 CX3CR1^GFP/GFP^).

### Genetic Deletion of CX3CR1 does Not Affect the Population of Utricular Macrophages

Macrophage recruitment to sites of tissue injury is mediated by a number of biological signals (Poon et al., 2014). One key modulator of macrophage recruitment and behavior is fractalkine (CX3CL1), a chemokine that is released by numerous cell phenotypes. Fractalkine interacts exclusively with the CX3CR1 receptor, which is expressed by macrophages, monocytes, and microglia (e.g., Ransohoff, 2009). Given the function of fractalkine signaling in other tissues, it was possible that disruption of fractalkine signaling (via deletion of the CX3CR1 receptor in CX3CR1^GFP/GFP^ mice) might affect the recruitment of macrophages into the sensory epithelium after hair cell injury. To resolve this issue, we compared the numbers of macrophages in utricles from Pou4f3–huDTR–CX3CR1^GFP/+^ (as reported above) with those observed at 7 days post-DT in utricles Pou4f3–huDTR–CX3CR1^GFP/GFP^ mice (*n* = 5). Nearly identical macrophage numbers were observed in both genotypes (**Figure 4C**), suggesting that fractalkine signaling does not serve a critical role in the injury-evoked recruitment of macrophages into the utricular sensory epithelium.

### Macrophages Within the Sensory Epithelium Phagocytose Hair Cell Debris

**FIGURE 5:**
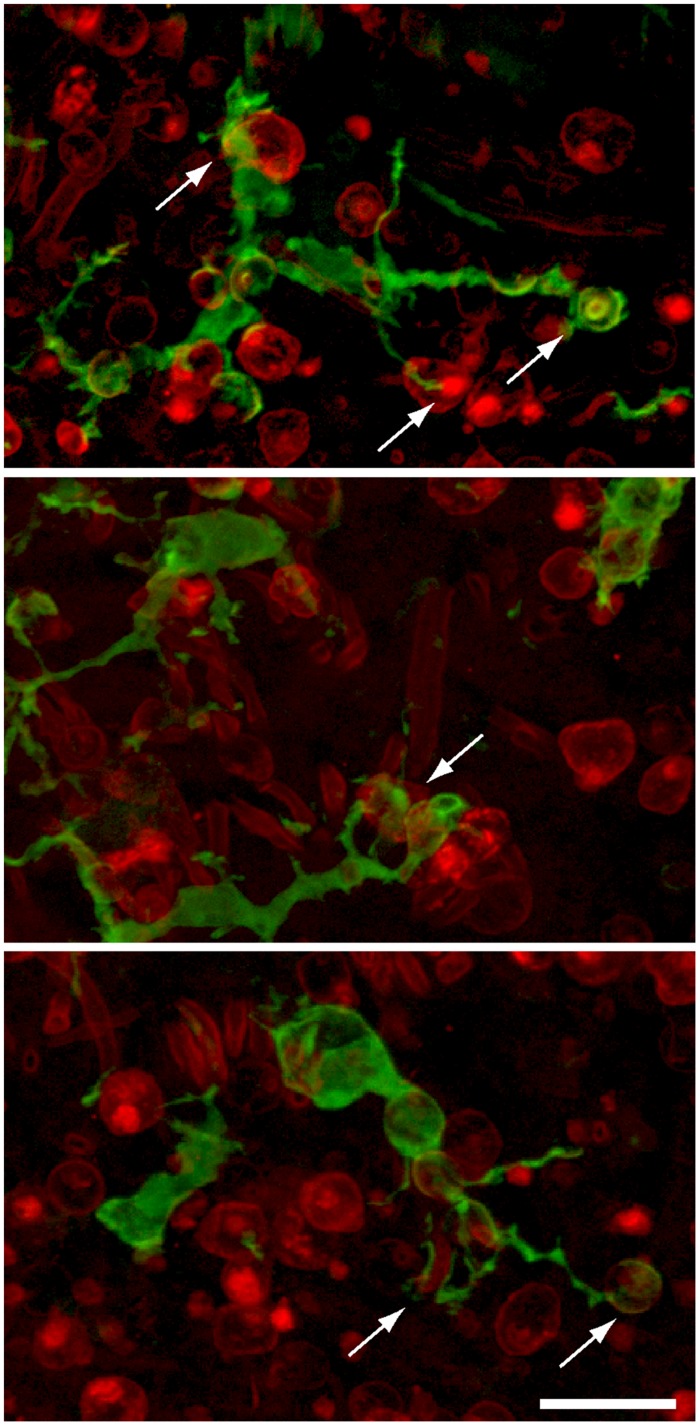
**Examples of GFP-expressing macrophages in the sensory epithelia of lesioned utricles.** Processes of macrophages (green) frequently appeared to contact (arrows) immunolabeled hair cells (red, otoferlin), suggesting that apoptotic hair cells may attract macrophage pseudopodia. Scale bar = 20 μm.

**FIGURE 6:**
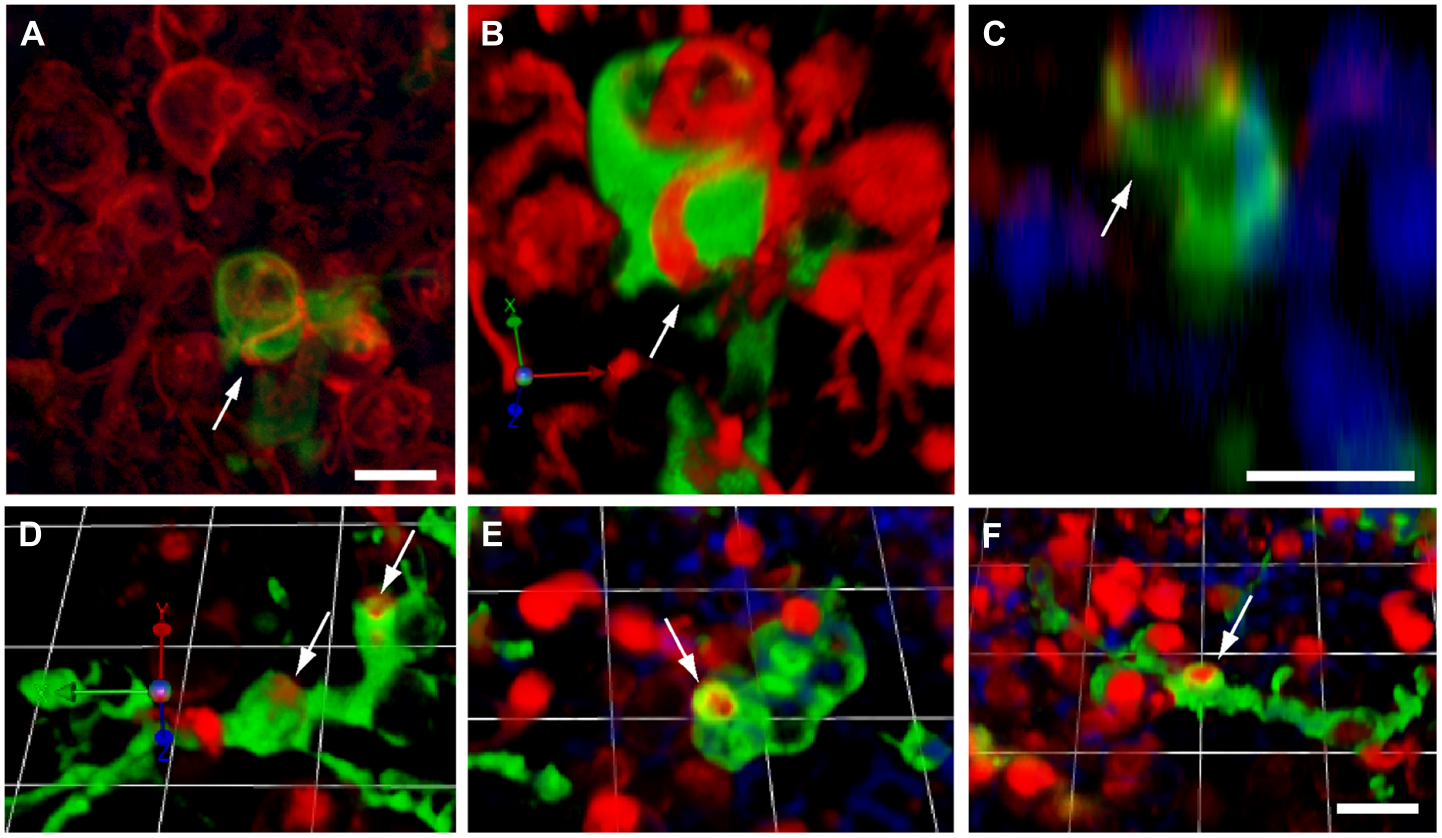
**Macrophage engulfment of hair cell debris.** Confocal images of labeled hair cells and macrophages often revealed hair cells that were apparently enclosed within the processes of macrophages (arrows). **(A–C)** Three different renderings of the same confocal stack, showing an otoferlin-labeled hair cell (red) being engulfed by a calyx-like phagocytic process of a GFP-expressing macrophage (green). **(A)**: Maximum intensity projection. **(B)** 3-D rendering of same image stack, showing the macrophage surrounding the hair cell. **(C)** z-plane slice from the image stack, centered on the engulfed hair cell, which is enclosed by the macrophage process (green). Cell nuclei are also visible (DAPI, blue). **(D–F)** Three dimensional renderings of images showing the engulfment of hair cell debris (red) by GFP-expressing macrophages (green). In all cases, phagocytic events are indicated by arrows. All scale bars represent 10 μm.

Removal of cellular debris after injury is essential for epithelial repair. In many epithelia, such debris clearance is mediated by a combination of nearby epithelial cells (‘amateur phagocytes’) and resident and/or recruited macrophages (‘professional phagocytes’). Prior studies have shown that macrophages are mobilized after hair cell injury, but it was not clear whether those cells actually engulfed hair cell debris. In the present study, confocal imaging of utricles from Pou4f3–huDTR–CX3CR1^GFP/+^ mice revealed numerous examples of GFP-labeled macrophages contacting labeled hair cells (**Figure 5**). Moreover, the incorporation of myosin VI and/or otoferlin-labeled material (i.e., hair cell constituents) into the GFP-labeled bodies of macrophages suggests that hair cell debris was being actively phagocytosed (**Figure 6**). This phenomenon was only observed in utricles from mice that possessed the huDTR transgene. In contrast, no phagocytic macrophages were observed in utricles taken from DT-treated wild-type (WT) mice.

### Supporting Cells Form Actin Baskets Within the Sensory Epithelium

**FIGURE 7:**
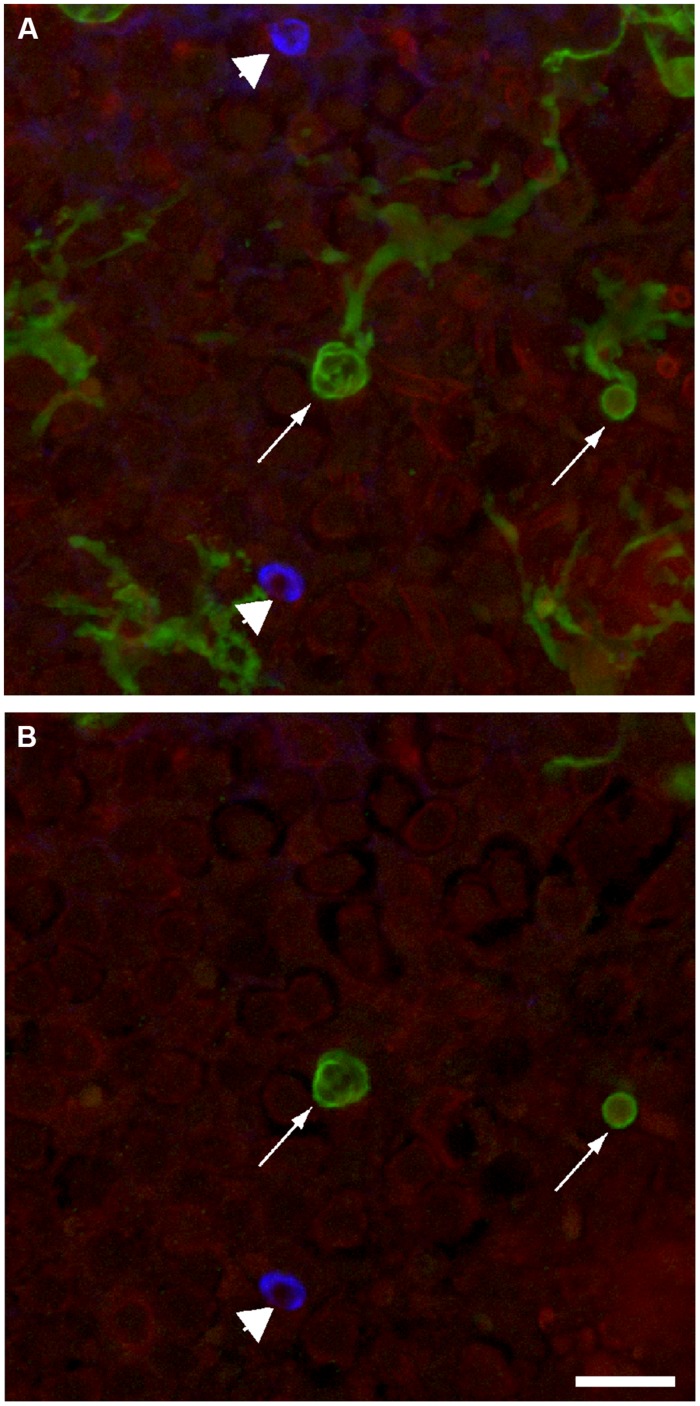
**Macrophages and actin basket phagosomes are simultaneously present in the injured mouse utricle.** Calix-shaped phagocytic processes of GFP-expressing macrophages were observed in all lesioned sensory epithelia (arrows). Phalloidin staining (blue) also revealed F-actin phagosomes in these same epithelia (arrow heads). These basket-shaped structures could be distinguished from macrophages by their strong phalloidin labeling (blue). **(A)** Maximum intensity projection of full confocal stack. **(B)** Single z-section through this stack, showing a cross section through macrophage processes (green) and an F-actin phagosome (blue). Scale bar = 20 μm.

The sensory organs of the inner ear use a variety of strategies for the clearance of apoptotic hair cells. For example, dying hair cells in the chicken cochlea are extruded through the lumenal surface of the sensory epithelium (Cotanche and Dopyera, 1990; Duckert and Rubel, 1990; Janas et al., 1995; Hirose et al., 2004; Warchol et al., 2012), while outer hair cells in the mouse cochlea are engulfed by nearby Deiters cells (Anttonen et al., 2014), and apoptotic hair cells in the maculae of mammals are removed by a combination of these mechanisms (e.g., Li et al., 1995). Time-lapse imaging studies of the chick utricle have demonstrated that hair cell death triggers nearby supporting cells to form basket-shaped actin structures, which phagocytose the remnants of dying hair cells (Bird et al., 2010). Formation of similar structures has been observed in the vestibular organs of mice (e.g., Ball et al., 2013; Furrer et al., 2014; Monzack et al., 2015). In the present study, we frequently observed actin ‘baskets,’ which labeled strongly with phalloidin and were confined to the lower strata of the utricular sensory epithelia. These putative phagosomes were easily distinguished from phagocytic processes of macrophages, which expressed GFP but did not contain significant levels of F-actin (**Figure 7**).

## Discussion

Hair cells can be lost from the inner ear after noise exposure, ototoxic injury, or as a consequence of normal aging. Because maintenance of the epithelial barrier is critical for separation of perilymph and endolymph, it is essential that the remaining cells quickly reseal the injured epithelium and remove cellular debris. These repair processes rely primarily on supporting cells, and appear to be mediated by different cellular mechanisms in the various hair cell epithelia of vertebrates. Specifically, supporting cells can undergo active and coordinated morphological changes, so as to extrude hair cell debris from the lumenal surface of the epithelium or to engulf such debris within the epithelium. Although macrophages are known to be present in the sensory organs of the inner ear, their role in epithelial repair and debris clearance is poorly understood. The present study characterized the behavior of macrophages in the mouse utricle in response to selective ablation of sensory hair cells. We found that the undamaged utricle possessed numerous resident macrophages, but that those cells resided in the stromal tissue below the sensory epithelium. However, hair cell death caused macrophages to enter the sensory epithelium, where they were observed to engulf hair cell debris.

### Epithelial Repair and Actin Remodeling after Hair Cell Lesions

Supporting cells in the vestibular organs of mammals possess thick circumferential bundles of filamentous (F-) actin (e.g., Burns et al., 2008). In the utricles of mice, these structures develop during the first post-natal month and their formation correlates with diminished ability for cell spreading, proliferation, and plasticity (Collado et al., 2011). It is notable that these thick actin structures are unique to the vestibular organs of mammals; cell–cell junctions in the maculae of fish, amphibians, reptiles, and birds all possess much thinner actin belts (Burns et al., 2013). The vestibular organs of non-mammalian vertebrates are also capable of quickly regenerating lost hair cells, while the regenerative abilities of the mammalian vestibular organs are much more limited (e.g., Warchol, 2011). Based on these observations, it has been hypothesized that the thick actin bands of the mammalian ear may be an important inhibitor of its regenerative ability (e.g., Burns et al., 2008, 2013). In light of this suggestion, it is notable that we found that the initial phases of epithelial repair in the mouse utricle appear mediated by much thinner actin bands at cell–cell junctions. Similar structures were reported by Burns and Corwin (2014), who used time-lapse imaging of the mouse utricle to characterize the early cellular events that occur after hair cell loss. The Pou4f3–huDTR mouse strain permits characterization of this process *in vivo* and over longer time spans. Our data suggest that the thin actin bands are only present at early times after hair cell loss; cell–cell junctions at later recovery times display much thicker actin structures (e.g., **Figure 2C**). We speculate that the (transient) presence of thin actin cables in the injured epithelium may correspond to a period of partial plasticity of supporting cell phenotype, i.e., a time during which those supporting cells with thin actin cables may be able to change their identity into new hair cells. Partial regeneration via phenotypic change has been demonstrated in the vestibular organs of guinea pigs and mice (Forge et al., 1998; Lin et al., 2011).

### Resident Macrophages in the Mouse Utricle

Our results confirm that the mouse utricle possesses a resident population of macrophages. In the uninjured utricle, resident macrophages appear to be confined to the stromal tissues below the sensory epithelium. Previous studies have suggested that many of these stromal macrophages are associated with small vessels or capillaries (Zhang et al., 2013). Although the functions of stromal macrophages are not known, the stromal is comprized of numerous cell phenotypes, many of which have been shown to interact with macrophages. For example, macrophages are known to release mitogens that stimulate proliferation of numerous cell types and they also participate in remodeling of the extracellular matrix (e.g., Chazaud, 2014). As such, it is likely that macrophages serve a number of distinct roles in the maintenance of inner ear stromal tissues. The notion is supported by the results of Warchol et al. (2012), who found that experimental depletion of macrophages from the avian cochlea resulted in reduced proliferation of the cells that reside immediately below the basilar membrane.

### Macrophages Phagocytose Hair Cell Debris

Prior studies have shown that macrophages are present either within or near the auditory and vestibular organs of birds and mammals (Warchol, 1997; Bhave et al., 1998; Hirose et al., 2005), but the precise function of otic macrophages has been enigmatic. In many tissues, resident macrophages quickly remove the ‘corpses’ of apoptotic cells, in order to maintain tissue integrity, and prevent inflammation (Stefater et al., 2011). Although it is reasonable to assume that otic macrophages serve a similar role, previous studies have failed to provide direct evidence for macrophage-mediated phagocytosis of dying hair cells. Moreover, experimental depletion of macrophages from the avian basilar papilla (cochlea) did not affect the removal of hair cell debris from the sensory epithelium (Warchol et al., 2012). Previously, the only clear demonstration of phagocytosis of dead hair cells by recruited macrophages has come from time-lapse imaging studies of hair cell regeneration in lateral line neuromasts of axolotl salamanders (Jones and Corwin, 1996). The present study unequivocally shows that identified (i.e., CX3CR1-GFP) macrophages can engulf the remains of dead hair cells. It is notable, however, that we also observed the presence of F-actin phagosomes within the lesioned utricle. Similar basket-like structures have been shown to engulf hair cell debris following ototoxic injury in the avian utricle (Bird et al., 2010) and in the mouse utricle (Monzack et al., 2015). Although a complete study of theses structures is beyond the scope of the present study, our findings suggest that hair cell debris may be removed from the mammalian vestibular organs via (at least) two distinct cellular mechanisms.

### Role of Fractalkine in the Regulation of Vestibular Macrophages

Finally, our use of knock-in CX3CR1-GFP transgenic mice allowed us to evaluate the role of fractalkine signaling in regulating macrophage activity within the vestibular organs. Fractalkine (also known as CX3CL1) is a chemokine that plays a variety of roles in the regulation of immune cells and in the interaction between the innate immune system and the nervous system (e.g., Ransohoff, 2009). Fractalkine is expressed on cells as a membrane-bound protein. Under certain circumstances, fractalkine can be proteolytically cleaved near the extracellular membrane surface, releasing a diffusible fragment that can function as a macrophage chemoattractant (e.g., Ishida et al., 2008). Following DT-mediated hair cell lesions, we found that macrophage entry into the utricular sensory epithelium was nearly identical in mice with intact fractalkine signaling (CX3CR1^GFP/+^) vs. those that lacked the fractalkine receptor (CX3CR1^GFP/GFP^). Because of inter-specimen variability, it was not possible to quantify and compare the total numbers of macrophages within the stromal tissue of CX3CR1^GFP/+^ and CX3CR1^GFP/GFP^ mice. However, visual inspection of those specimens did not reveal any qualitative differences in macrophages between those two genotypes. Notably, a prior study has shown that genetic deletion of CX3CR1 leads to increased macrophage numbers in the ototoxically injured cochlea (Sato et al., 2010). Given these findings, we believe that the present data should be interpreted with caution. Specifically, our data suggest that lack of fractalkine signaling does not impede macrophages from entering the utricular sensory epithelium after selective hair cell injury. We speculate instead that other diffusible molecules that are released from dying hair cells and/or their adjoining supporting cells (e.g., ATP – Gale et al., 2004; Lahne and Gale, 2008) may attract macrophages to sites of hair cell injury. Future experiments will be necessary to resolve this issue.
